# The Use of Organic Acids (Lactic and Acetic) as a Microbial Decontaminant during the Slaughter of Meat Animal Species: A Review

**DOI:** 10.3390/foods10102293

**Published:** 2021-09-28

**Authors:** Davies Veli Nkosi, Johan Leon Bekker, Louwrens Christian Hoffman

**Affiliations:** 1 Department of Environmental Health, Tshwane University of Technology, Pretoria 0001, South Africa; bekkerjl@tut.ac.za; 2 Department of Animal Sciences, University of Stellenbosch, Private Bag X1, Matieland, Stellenbosch 7602, South Africa; louwrens.hoffman@uq.edu.au; 3 Centre for Nutrition and Food Sciences, Queensland Alliance for Agriculture and Food Innovation (QAAFI), The University of Queensland, Health and Food Sciences Precinct, 39 Kessels Rd., Coopers Plains, Brisbane 4108, Australia

**Keywords:** abattoir, illegal slaughter, wild ungulate

## Abstract

Wild ungulate species provide a much-needed protein source to many communities in developed and developing countries. Frequently, these game meat animals are slaughtered, and the meat is unknowingly contaminated by microorganisms and released to the unsuspecting public. This review investigates the global usage of organic acids (lactic and acetic acids) as microbial decontamination strategies during slaughter. The results show that there is a more open-minded approach to adopting possible decontamination plans as a tool to improve meat safety during slaughter. Developed countries continue to adopt these strategies, while developing countries are lagging behind. While decontamination of carcasses can lead to a reduction of microbial load on these carcasses, this strategy must not be seen as a replacement of hygiene management during the animals’ slaughter.

## 1. Introduction

Food microorganisms can be found throughout meat processing plants. This suggests and highlights the importance of monitoring, controlling and ensuring that these organisms, especially pathogenic organisms, do not contaminate carcasses during slaughter [1,2]. Generally, carcasses are free of microorganisms when slaughtered in a hygienic manner, and the meat derived from the animal slaughtered remains safe and healthy after slaughter [3]. However, the processing of game/wild animals slaughter could cause meat contamination during killing and dressing in the field or slaughter at the abattoir from various sources such as faecal material, paunch and hide, processing tools and equipment, the facility, human contact, the environment (air, water, etc.), and carcass-to-carcass where insufficient space is left between already dressed carcasses and undressed carcasses on the slaughter line [4,5]. Under normal circumstances, there are two game-meat animal-killing methods employed in the field: killing with a single projectile shot or killing with a shotgun (utilising numerous pellets). These in-field killing processes coupled with exsanguination (neck slitting and/or thoracic sticking) and evisceration utilising spear cuts have the potential to leave open cuts on the body, thus exposing the meat to microbial contamination [6]. As stated by [7], slaughter processes, if not well monitored, may result in dangerous microorganisms being transferred from one carcass to another. While it can be argued that this type of contamination can be avoided by proper training of slaughter operators, meat inspection and the general abattoir hygiene application by personnel, total elimination of microorganisms cannot be achieved. It is noted that while carcass trimming on observable contaminated surfaces is mandatory during meat inspection, this practice could be seen as meat wastage and throwing away of good protein that is usable [8]. In response to this challenge, many researchers expressed the possible effective use of decontamination strategies to remove organisms that could be present on carcasses before being released to consumers [9,10,11]. The use of organic acids on surfaces of meat products or meat during processing has been investigated in the past [12,13]. Investigations have been done on the efficiency of reducing microorganisms by introducing organic acids producing bacteria on surfaces of meat products [14,15]. Most of the studies conducted were on processed meat products and poultry carcasses and a few were on red meat carcasses, and the usage of organic acids on fresh carcasses during slaughter still needs to be further investigated [13,16,17,18,19]. In describing microbial decontamination, Han et al. [19] state that these processes expose food products or carcasses to a specific agent, or a combination thereof, such as steam, chlorination, trisodium phosphate, pulsatile light exposure, pulsed electric fields or ionizing radiation, and organic acid solutions with the aim of reducing the amount or concentration of the microorganisms. In other instances, hot steam is used as a form of decontamination [20,21]. While it can be confirmed that decontamination can be used to effectively reduce the number of microorganisms, it must be viewed as a meat safety strategy to be added to existing programs of hygiene, such as the use of a two-knifes system during animal slaughter and dressing, prevention of animal hide from coming into contact with already dressed sides and meat inspection/trimming to physically remove visible contamination already implemented during slaughter [22]. Reference [23] note that various processes of meat decontamination are not generally accepted across the globe. For example, the United States of America (USA) has effectively implemented a carcass decontamination plan, whilst some European countries do not fully endorse the use of decontaminates as a form of improving the safety of meat products, with an exception of lactic acid and potable (chlorinated) water. A few developing countries approve the use of decontamination agents; this is mainly caused by a lack of data or information on the implementation of the decontamination plan in these countries. This situation is no different in South Africa, where only chlorinated water can be used to rinse fresh carcasses after slaughter before chilling; no other methods of decontamination are approved yet. However, the obligation lies with industry to prove the efficiency and effectiveness of a decontamination system before it can be approved to be used in the meat industry [24]. Given the ever-changing environments and the ever-growing demand for meat and thus demand for slaughter, measures that can benefit the industry and at the same time improve the safety of a specific product must be developed and implemented [23]. Many researchers have identified citric, lactic and acetic acid as possible organic acids that can be used to reduce some types and numbers of microorganisms in wild ungulate species meat. As a perishable product, meat of animal origin also carries a significant number of microorganisms. These organisms include but are not limited to *Salmonella, Campylobacter* and *Escherichia coli* and some strains of *Listeria monocytogenes* [7]. These microorganisms and many others must be identified, monitored and controlled in a food processing plant such as an abattoir [25].

## 2. Decontamination Plans for Game Meat Animals during Slaughter

While there are many decontamination plans and systems adopted in food processing, the situation is different at slaughter plants or abattoirs, where fewer decontamination plans may be used [18]. These include a combination of water used to wash carcasses and chilling. The chilling effect that the residual water may have during evaporative chilling can help to reduce the number of microbes on the carcass surfaces. Other strategies include the use of chlorinated water, organic acids such as lactic, acetic and citric acids, and hot steam [10,12,19]. The main challenges of these interventions are as follows [9,20]:

- Most decontamination strategies may change the appearance and texture of a product.
- Specific concentrations must be maintained to ensure that they do not alter the texture of meat products.
- There is a lack of sufficient data or information on the usage of different decontamination regiments on carcasses whilst still maintaining the quality of the product.
- There can be a large cost of implementing a decontamination plan on top of the general hygiene prescripts that must be followed during animal slaughter at an abattoir.

According to [21], the usage of specific organic acids at lower concentrations can achieve the desired effect of reducing or killing microorganisms without influencing the quality, texture, smell and appearance of meat. The important factors to be considered are the time of application, the simplicity of the process, the availability of the decontaminant and the concentration of the acid. Reference [8] note that microbial decontamination strategies or plans should be used as a secondary measure of limiting micro-organisms on carcasses and must not replace the general hygiene application and good manufacturing practices employed by meat processors with respect to hygiene requirements.

This clearly implies that if slaughter is done correctly with proper hygiene management, there should not be a need to do any additional decontamination [22]. For the purpose of this review, the usage of acetic acid and lactic acid was examined to determine their usage as a microbial decontaminant during the slaughter of game meat animals. The selection of these acids was influenced by their availability and usage in food processing and the fact that they are also organic in nature and thus more acceptable to food processors and authorities [1,8,22]. It must be emphasized that in South Africa, no form of carcass decontamination/treatment is yet approved. Forthe benefit of meat safety and improving the principles of hazards control during slaughter, the potential use of organic acids as forms of decontamination plans should be investigated.

### 2.1. Organic Acid Usage

Treatment of carcass surfaces with organic acids can have a positive result in the inhibition of microbial growth [11,21]. This is mainly due to the fact that organic acids tend to promote the disruption of the proton motive force (PMF) created by microorganisms on the cell surface. This disruption subsequently leads to the creation of an unfavorable environment for microorganisms to thrive [11]. As confirmed by [10], organic acids tend to influence the microbial activity on the treated surfaces such as fresh meat; this then leads to the increase in the pH of the surface to a level intolerable by general microorganisms. It is through these processes that an organic acid is able to reduce or kill microorganisms on treated surfaces.

### 2.2. Lactic Acid Treatment

Lactic acid (LA) is a naturally occurring acid and is used effectively by the food industry during food processing. Another source of LAs is food waste, particularly of dairy products and especially sour milk [24]. The reason behind this widespread usage includes its ability to mix well with water and its anti-microbial capabilities [11,21]. It is also used as a preservative in food products and in cleaning and sanitation of food and food contact surfaces [25]; LA has been effectively and extensively used as a decontaminant in the food industry for general microorganisms, some of which are pathogenic, such as *Salmonella* and *Escherichia coli* [26].

### 2.3. Acetic Acid Treatment

Acetic acid (AA) is another organic acid extensively used in the food industry. In addition to its preservation capabilities, AA can be used to kill or reduce other microorganisms of interest in food or meat products [27,28]. Researchers have argued that while the use of AA in its concentrated form could be beneficial in reducing microorganisms, its strong pungent smell could be a deterrent to its usage on fresh carcasses [10,29]. This could be overcome by mixing it to less than 4% or lower in concentration and spraying this mixture onto areas prone to contamination such as the neck area around the bleeding cuts, bullet entry points in the case of body kill on game meat animals, first and second spear cuts’ areas, hind legs opening lines, evisceration points and the brisket areas [30]. Researchers [10,11,21] have identified citric, lactic and acetic acids as possible organic acids that can be used to reduce the number of microorganisms in red meat. It is the aim of this review to critically evaluate the use of organic acids in raw game meat and game meat products with the purpose of their use as microbial decontamination agents.

## 3. Materials and Methods

**Figure 1:**
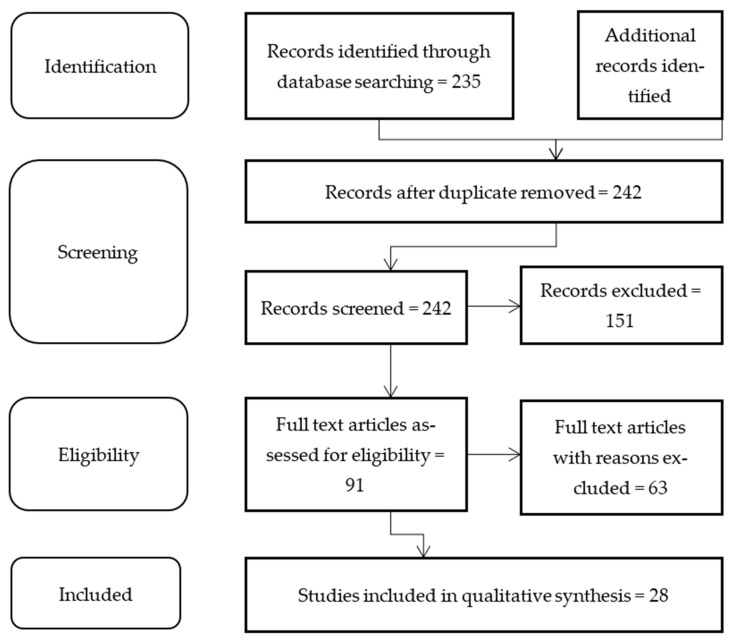
Methodology of search followed during the review process.

This review was compiled from English scholarly literature as sourced from Google Scholar; Science Direct; PubMed between 2011 and 2021. The procedure used to search included entering the following key terms: “Carcases OR Game meat OR Wild meat AND Decontamination OR Lactic Acid OR Acetic Acids OR Carcass wash AND Africa OR Europe OR South America OR North America OR Asia OR Australia OR Oceania OR Antarctica”. Grey material from web pages of the Codex Alimentarius (www.fao.org) (Accessed: 28 June 2020) was also searched for the latest update regarding the implementation of decontamination plans by food authorities for fresh carcasses at abattoir levels across the globe (Table 1). Figure 1 depicts the search and review methodology of the literature pursued on organic acid usage as a decontaminant of fresh meat across the world as per the PRISM diagram adapted from [26].

Records with no specific reference to carcass decontamination by acetic acid, lactic acid or organic acid; studies in languages other than English; and postgraduate theses were excluded from this study as they did not relate to the objective of the study. An overview of studies conducted between 2011 and 2021 globally is presented in Table 1.

## 4. Results

**Figure 2:**
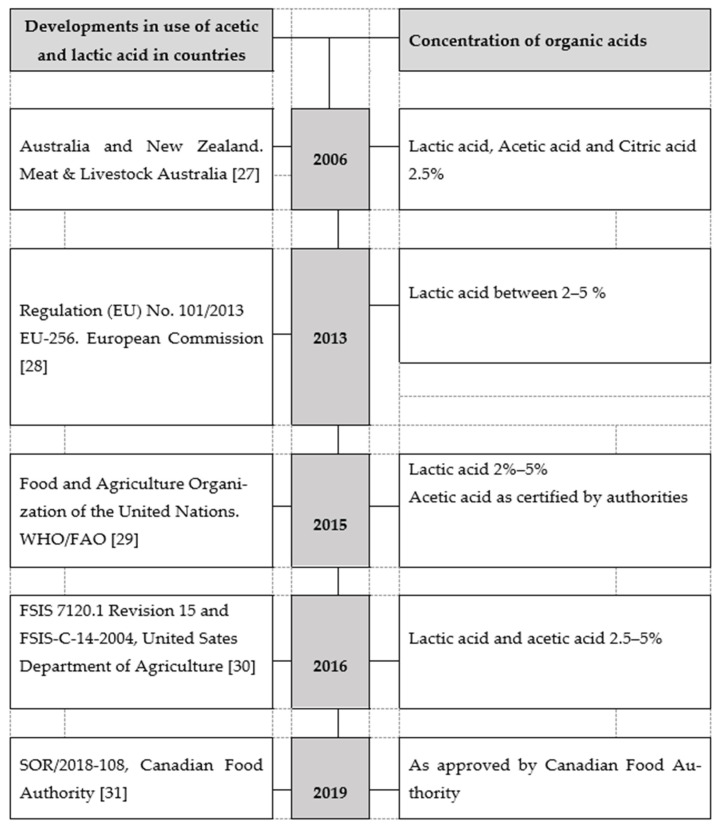
Regulations and guidelines on the use of acetic and lactic acid to decontaminate carcasses globally. Note: The legislation and guidelines, or standards are from the inception of any relevant legislation. Existing and current regulations or guidelines are included from the date they became applicable or date of release. Some of the countries started developing these guidelines as early as 2006.

In an “any time” search on Google Scholar using the above-mentioned keywords, the first recommendation on the use of organic acids to decontaminate slaughter animal carcasses was as early as 1990 in Egypt [31]. It is evident that the concept of carcass decontamination is not new in the world. Strides have been made to improve and make these carcass decontamination plans applicable to wild ungulate species, especially in developed countries. Figure 2 provides a timeline of legislative advances and guidelines globally from 2006 (first year of detection during study) to the present. Evaluating country-to-country, Table 1 presents the frequency of acetic and lactic acids studies conducted between 2011 and 2021. The numbers of studies conducted were as follows in the United States of America (10), Canada (2), Spain (2), Serbia (1), Egypt (2), Switzerland (1), Australia (1) Japan (1), Greece (1), Sri Lanka (1), France (1), Pakistan (1), Romania (1), Turkey (1), Mexico (1), and Singapore (1). No information could be found on research done in any of the other countries of the world.

Figure 2 provides a summary of regulations and guidelines on the use of acetic and lactic acid to decontaminate carcasses globally.

While meat decontamination is continuously investigated globally, by the end of 2020, few countries had developed guidelines or regulations that dealt with carcass decontamination during slaughter [27,28,29,30,32]. As noted by [31], developing countries typically rely on the guidelines of the Food and Agriculture Organization (FAO) when applying carcass decontamination programs. However, the responsibility of approving such a plan belongs to the country where such a program is implemented.

**Table 1:** Summary of the use of acetic and lactic acid to decontaminate carcasses derived from research (2011–2021). The order of presentation in ascending order of publication date.

Country	Aim	Product Investigated	Experimental Conditions	Study Findings and Recommendations	Reference *
United States of America	To compare spray washing at 55.4 °C of a 2% levulinic acid with lactic or acetic acid for decontamination of pathogenic bacteria inoculated onto meat surfaces and their residual protection against later growth of pathogenic bacteria	Red meat and poultry	Lab experiment on inoculated red meat and poultry plates Comparison of 55.4 °C with 2% of levulinic acid	Lactic acid provided the greatest efficiency in decontamination out of the three acids. Acetic acid provided the second-best microbial growth inhibition. Levulinic acid did not provide as effective decontamination as lactic acid. Decontamination was between 0.6 to 1 log/cm^2^ compared to controls, which was a no-wash treatment of meat surfaces.	[33]
Switzerland	To examine antibacterial activity of LA, AA and steam as decontamination treatments for cattle hides and beef carcasses	Beef	Literature studies on possible decontamination of beef hides and carcasses during slaughter.	A combination of LA and AA during application yielded the desired results of microbial reduction compared to a single OA application on beef carcasses. The general reduction for indicator microorganisms ranged between 0.7 and 4.9 logs.	[34,35,36]
United States of America	To examine mechanisms of reducing contamination by *C. jejuni* in broiler carcasses that were vaccinated with Lactobacilli as chicks.	Poultry	Broiler chickens were inoculated with *lactobacilli* on the day of hatch, day four, day fourteen and day twenty-one after hatch.	The production of organic acids by “Lactobacilli” can be effectively used to reduce a load of pathogens in poultry carcasses. The use of *lactobacilli* in live chicks can be adopted to control the levels of *C. jejuni* that may be present in carcasses. Organic acids such as LA and AA must be included in the future development of competitive decontamination strategies on carcasses at abattoirs.	[37]
Serbia	To investigate possible interventions of controlling Salmonella contamination during poultry, beef and pig slaughter	Poultry, beef and pork	Literature review on the benefits of decontaminations of poultry, beef and pork carcasses	A combination of LA and AA at suitable concentrations of between 2 and 5% could be used effectively to reduce numbers of microorganisms. Pre-skinning decontamination of carcasses should be investigated to aid the decontamination plans during slaughter. Consider using LA and AA in combination with hot water of between 72 °C and 85 °C or steam between 82 °C and 99 °C. A concentration of 2% LA can reduce up to two folds of Salmonella on pig carcasses.	[38,39,40]
United States of America	To determine the effectiveness of eight antimicrobial compounds including LA and AA in a laboratory.	Beef surfaces	Small beef processing plants 2% LA used on beef surfaces by pressurized handheld OA spray equipment	Decontamination plans using both LA and AA are affordable and simple to apply at small processing plants. The combination of different treatment plans such as use of chlorinated water and organic acid at different stages of the slaughter program should be investigated.	[41]
Turkey	To compare the inhibitory effects of various decontamination agents at different OA concentrations on *Listeria monocytogenes* contaminated raw beef samples.	Beef	Beef samples contaminated with *L monocytogenes* were exposed to different concentrations of LA 1–2%, AA 0.1%	Two percent LA provided the most effective inhibition of *L monocytogenes.* Inhibitory to the effective implementation of a contamination plan includes selection of proper OA, pressure and solution temperature.	[42]
Mexico	To investigate microbial adaptation to OA as antimicrobials to control Salmonella in meat and poultry products.	Poultry	Literature review on the use of the OA to control Salmonella in meat and poultry products	*Salmonella* spp. could develop an adaptation to LA and other OA. OA treatments are not optimal, and sub-lethal conditions can induce the development of adapted or resistant strains to OA.	[43,44]
Canada	To investigate microbial decontamination of raw and ready-to-eat meats using OA	Raw and ready to eat meat	Literature review discussing the adoption of new technology adoptable to reduce microorganisms in fresh and processed meat	Uncontrolled implementation of decontamination plans could hinder the appearance and taste of meat products. Appearance degrading treatments can be applied to manufacturing meat that will be further processed.	[44,45]
Singapore	To establish different intervention technologies ensuring microbial safety of meat	Raw meat	Literature review on possible safer meat-producing strategies adoptable by the meat industry	Solutions of LA and AA (1% to 3%) are commonly used successfully for beef and lamb. These OA may be applied as single mixtures or a combination in carcass wash facilities. Steam or water sprays could be used as an applicator.	[46,47,48]
Spain	To investigate effective control and treatment plans for *Campylobacter* in abattoirs.	Poultry	Literature review on existing controls adoptable by EU countries for poultry slaughter	A combination of hot water, LA, acidified sodium chlorite or trisodium phosphate resulted in reductions of between 50 and 90% of microbial growth. A two log10 reduction would lessen the risk to humans by more than 90%. Application of these interventions during slaughter could totally eliminate *Campylobacter* in meat.	[49,50]
United States of America	To establish the efficiency and effect of different concentrations of LA, AA, citric and propionic acid dipping solutions on bacterial contamination of raw chicken skin	Poultry	Chicken skin dipped in 10^8^ cfu/mL of salmonella, *E. coli* and listeria for 30 s then treated with different concentrations of OA ranging between 0.2, 0.4, and 0.6%	The concentration between 1 and 4% of LA and AA could be used to reduce the number of spoilage organisms and improve food safety properties of raw poultry skin.	[51]
United States of America	To investigate the survival and adaptation of *Salmonella* spp. when subjected to acidic conditions on carcass surfaces.	Beef and porcine	LA and AC at a pH ranging between 4.0, 5.0 or 6.0 incubated for between 6 to 48 h at 37 °C	*Salmonella spp*. can develop tolerance to LA and AA, especially at pH 5.0 and 6.0. The developed or implemented plan must consider different microorganisms and different types of facilities.	[52]
Greece	To analyse carcass decontamination strategies employable in slaughterhouses: a review	Meat animal carcass	Literature review of possible carcass decontamination plans employable during slaughter	The selection of a type an OA, its concentration, application time and pressure should influence its efficacy to reduce microorganisms during slaughter at small facilities.	[35,53]
Sri Lanka	To investigate the effect of natural compounds and acids on *Salmonella typhimurium* in broiler chicken meat	Poultry	Chicken samples contaminated with *salmonella at 1%* solution of LA, AA and CA was treated for 30 s	Natural compounds (citric, acetic and lactic acids) showed a 20% greater reduction of colony count in broiler chicken compared to chemical compounds. OA has an effect on *S. typhimurium* and therefore can be used for the decontamination process of poultry meat carcasses during slaughter.	[54]
United states of America	To determine the bactericidal activity of lactic acid (LA), levulinic acid (LV) and sodium dodecyl sulfate (SDS) applied individually and in combination with Shiga toxin-producing *Escherichia coli* (STEC) under laboratory conditions	Beef cuts-experiments on trimmings	LA applied at a concentration of 3% to determine its efficiency at 21 °C to kill *Escherichia coli* (STEC) on beef trimming	LA, LV and SDS substantially reduced microbial contamination on beef trimmings of both pathogens, with no detectable *E. coli* O157:H7 or *Salmonella typhimurium* (<5 CFU/cm^2^) on beef trim pieces treated with lactic acid (LA), levulinic acid (LV), and sodium dodecyl sulfate. Meat or temperature played a big role in influencing microbial load reduction.	[55]
France	To investigate lactic acid bacteria (LAB) and their controversial role in fresh meat spoilage	Raw meat	Literature review of the beneficial uses and non-beneficial effects of LAB on raw meat products surfaces	LAB species applied on fresh meat may be beneficial by outgrowing the rest of the microbiota and improve the safety of the meat product. Some strains of LAB may lead to rapid spoilage of meat products, thus affecting the quality and subsequent shelf life of fresh meat products. The final evaluation and approval of an effective LAB strain to be used on carcasses must be done this is subject to sensorial tests.	[56,57]
United States of America	To investigate antimicrobial formulations and sanitation methods for meat and poultry product processing.	Poultry	A review of new trends of decontaminating meat and poultry processes	The use of chlorine only as a decontaminant on poultry products is not effective. A combination of LA and AA with chlorine at different intervals could reduce the number of micro-organisms.	[58,59]
Pakistan	Postharvest intervention technologies for safety enhancement of meat and meat-based products; a critical review	Beef	A critical review of trends followed to control post-slaughter pathogens	OA solution, mainly AA, LA or citric acid at 1.5–2.5%, was adopted for decontamination of beef carcasses at slaughter facilities. A hurdle technology should be adopted during slaughter.	[4,60]
United States of America	To evaluate the ability of a bromine-based antimicrobial lactic acid (LA) and peroxyacetic acid (PAA) applied in a final carcass wash to reduce non-pathogenic *Escherichia coli*.	Bovine	Beef carcasses inoculated with 6 log CFU/cm^2^ of *E. coli* biotype at an abattoir Concentrations of LA 2–5%	The series of interventions of LA and PAA in a complete system was effective against inoculated and non-inoculated microbial populations on beef carcasses in a commercial beef harvest operation.	[61]
Spain	To test the efficiency of lactic acid concentrations on the reduction of microbial load yet minimally impact the colour and sensory characteristics of beef	Beef	Beef products were treated with concentrations of LA ranged between 2 and 5% to determine sensory changes of beef products	Lactic acid has recently been approved in the European Union as a beef decontaminant during slaughter. LA at 2% to 5% might improve the microbiological quality of beef, as compared to untreated meat. Sensory changes may be present on beef products.	[62]
Canada	To investigate possible pathogens reduction strategies employable at the primary production level especially in relation to multi drug-resistant strains	Raw meat	Literature review on possible pathogens reduction plans for meat processing plants	Interventions should be aimed at primary animal health care, good hygiene practices and training. The reliance on end-process decontamination should be limited to few organic acids such as LA and AA. Such treatments are subject to approval.	[63]
Romania	To assess the efficiency of organic acids LA, AA and citric acid in different concentrations on pathogens such as *Salmonella*, *Listeria* and *Escherichia* on beef.	Beef	Concentrations of LA, AA and CA ranged from 1–3% at a volume of 25 mL Inoculation with *Salmonella enteritidis, Escherichia coli* and *L monocytogenes*	Among the OA, the most efficient was LA, followed by AA. Citric acid CA remained less efficient in reducing microorganisms. The greatest reduction in microorganisms was determined at a concentration of 3% by LA.	[27]
Japan	To evaluate the effect of LA with and without organic material at various post-treatment recovery times on the heat resistance of *Listeria monocytogenes.*	Bovine products	Lactic acid concentration of 0.5–5 % was used to determine OA’s effectiveness to kill strains of *L monocytogens* in a laboratory experiment of inoculated beef solution	Influence of LA and post-treatment recovery time on the heat resistance of *L. monocytogenes*. The need to pay attention to the combination of acid treatment and subsequent hygiene application during manufacturing processes or slaughter processes to minimise contamination.	[18]
Egypt	To test the antibacterial effect of lactic acid (LA) and acetic acid (AA) on sheep carcass surface after 20 min of spraying.	Sheep carcasses	Concentrations of OA 1, 1.5 and 2 % used to decontaminate aerobic microorganisms on sheep carcasses after slaughter	Spray treatments using three concentrations (1, 1.5 and 2 %) can be effectively used on fresh carcasses to reduce aerobic plate count, *Enterobacteriacae* count, coliform count and Staphylococcus count of fresh sheep carcasses.	[64]
United States of America	To investigate the effectiveness of organic acids (LA) on *Salmonella* ssp. reduction on ground beef.	Beef	Beef trimmings inoculated with 3.5 log of salmonella strains after grinding and exposed to OA.	The applications of LA at 5% and peroxyacetic acid at 600 ppm on beef trim did not decrease *Salmonella* populations in ground beef.	[65]
United States of America	To establish the interactions of organic acids (LA and AA) with *Campylobacter coli* from swine	Red meat	Measure the effective pH of OA to inhibit the growth of *111 C. coli* strain on meat surfaces	OA carcass wash may not provide the expected elimination of surface bacteria if the concentration levels of the dissociated OA used are not carefully controlled and a required pH is achieved.	[66]
Australia	To investigate meat safety risks for the Australian red meat market	Red meat	Technical report of current practices and regulations accepted by Australia red meat industry (a technical study)	Interventions that are commonly used in Australia include a combination of trimming, hot water, steam, LA and AA.	[67,68]
Egypt	To investigate the effect of LA, AA and trisodium phosphate (TSP) spray on the microbiological population.	Beef carcasses	Beef carcass obtained after slaughter. OA concentrations, LA 2%, AA 2% and TSP 12%	LA and AA can be used effectively for the purpose of decontamination in abattoirs during slaughter. TSP was less effective in decontamination when used alone.	[69]

* Some of the original source papers predate 2011. LA = Lactic acid. AA = Acetic acid. OA = Organic acid.

## 5. Discussion

This review has shown that while extensive research has been done on meat animals, there is still more to be done in the application of organic acids (OA) in developing countries such as South Africa. Globally, South Africa is regarded as a diverse wild meat-producing country. A large proportion of these wild animals cohabitate with livestock, a situation that could lead to cross-contamination of microorganisms between these two groups of animals [22,70]. In fact, antibiotic-resistant microorganisms have been found between wildlife and farmed species in South Africa [71,72]. It is therefore surprising that no published works on adapted decontamination plans from livestock abattoirs for wildlife are available. While the demand for meat continues to grow, advances in improved slaughter, decontamination methods and legislation to produce safer meat products have been slow in developing countries and Africa as a continent [3,49]. This situation is no different from that of the consumption of (microbially safe) wild meat animals. In general, it can be accepted that the use of OA as a form of a microbial decontamination intervention during the slaughter of game meat animals can be adopted.

The decontamination of carcasses by organic acids such as LA and AA is reliant on the following factors: uniform application of the OA over the carcass at appropriate intervals, concentration of the OA and pH, temperature of the acid and of the carcass being treated, pressure of the application, contact time and a combination of decontamination strategies. Industries must decide on the best OA application plan to ensure an effective and efficient decontamination response. Reference [33] confirms that the majority of microorganisms do not survive in stronger acids; however, the concentration of these acids should be such that they are efficient whilst still retaining acceptable sensory qualities of the treated meat portion [21,73]. This is confirmed by [40], who noted that when higher concentrations of organic acids are used, secondary rinsing with potable water may be needed to remove the acids following their application. This is done to balance the sensory qualities of treated meat products. This could prove to be a challenge in wild animals as they are normally slaughtered in field-abattoirs where potable water is scarce [74].

The optimal pH values for an effective OA range between 2.5 and 3; while this could be seen as viable and possible to achieve, studies have also highlighted that the contact time between an OA and a surface is important in the reduction of microorganisms [55]. While the killing of microorganisms could occur within seconds, it is important to ensure that sufficient time between 2 and 10 min is observed to ensure sufficient treatment [65,75]. Additionally, the temperature of the acidic solution and the temperature of the surface or product could determine the efficiency of a decontamination plan. As noted by [31,76] an increase in the temperature of the OA solution and the application of OA while the carcass temperature was still warm led to the desired results of microbial reduction [38,73]; this could be enhanced by applying the OA solution in the form of steam between 50 and 55 °C while the carcass is still warm during the slaughter process [10,21]. This suggestion might be applicable in formal abattoirs where heating systems and potable water are readily available, but in field abattoirs where wild animals are frequently processed, heated water is limited; most decontamination systems make use of concentrated chlorine solutions [74].

Reference [77] explain that the influence of the steam solution temperature and carcass temperature can also be enhanced by increasing the pressure of the applicator; increasing the application pressure of the organic acid applicator achieved a log reduction of between 1 and 2 logs of aerobic microorganisms in raw meat [34]. This was confirmed by [78,79] where higher pressure yielded better microbial decontamination of *Campylobacter* on carcass surfaces. It can be accepted that there is no perfect system(s) that could guarantee the total elimination of microorganisms on meat surfaces during slaughter. It is important to ensure that the best possible microbial treatment interventions or a combination of interventions are adopted during slaughter. This is generally termed the hurdle technology approach. Hurdle technology can be described as using different microbial hurdles to achieve the basic condition of effective decontamination of carcasses during slaughter [80]. This technology could be applied effectively by using a combination of interventions such as improved hygiene application, improved meat inspection, trimming of suspected carcases/areas and introduction of organic acids in appropriate concentrations and pH on a carcass and swift chilling of dressed carcasses.

The OA could be applied as a single solution or in combination with other organic acids in the form of steam and/or water at varying pressures [66,81]. The development and use of OA in meat decontamination strategies must be done in a responsible and controlled manner to prevent the development of resistant strains of microorganisms.

References [71,72,82,83], as well as the study by [79], showed that prolonged usage of specific acids in a food facility may facilitate the development of resistant strains. As there are currently insufficient data available on microorganisms resistant to OAs, this situation should be monitored and controlled. Authors should discuss the results and how they can be interpreted from the perspective of previous studies and of the working hypotheses. The findings and their implications should be discussed in the broadest context possible. Future research directions may also be highlighted.

## 6. Conclusions

While decontamination is intended to remove or reduce the concentrations of microorganisms on carcass surfaces, the role of hygiene during slaughter should never be compromised. It is clear that given advancing research, more and more ideas for decontaminating game meat will be developed. Similarly, these ideas must be investigated for their effectiveness as well as usability, given that there are few factors that may determine the efficiency of these treatments, including: (1) temperature of the mixture, (2) temperature of the carcass, (3) time contact allowed settling on the carcass surfaces, (4) type of meat surface and (5) the pH of the carcass. These systems should include microbial monitoring at farm areas, minimisation of stress during the killing of animals, training of slaughter operators on hygiene application during slaughter and application of an accepted decontamination plan. It is important to note that in other instances, a combination regime of OA could be useful in the fight against microorganisms in game meat. In the South African context of the game meat industry, it is important that more investigations at abattoir or slaughter levels are conducted to determine the practical application of these treatments/methods (e.g., using heated/steam mixtures). It can be accepted that while there are many OAs used for carcass decontamination interventions, lactic acid (LA) and acetic acid (AA) have been adopted predominantly in meat products and carcasses in developed countries. Most of these interventions are product or species-based and cannot be used broadly for all meat animal species. There is clear evidence that LA and AA can reduce microbial colonies in beef, poultry, porcine and other meat products; what remains in question in general is their application in game meat and as part of an alternative process within a food safety management plan for the game meat industry.
